# Telemedicine for educating parents or caregivers for postoperative care of pediatric patients: a systematic review

**DOI:** 10.3389/fpubh.2025.1606211

**Published:** 2025-07-30

**Authors:** Grecia Victoria Vivas-Colmenares, Jose Ruben Ramírez-Iglesias, Ana M. Martínez-Pérez

**Affiliations:** ^1^Health Sciences Faculty, School of Medicine, Universidad Internacional SEK (UISEK), Quito, Ecuador; ^2^International Doctoral School, Universidad Rey Juan Carlos, Madrid, Spain; ^3^Department of Communication and Sociology, Faculty of Communication, Universidad Rey Juan Carlos, Madrid, Spain

**Keywords:** pediatric surgery, follow-up, telemedicine, post-operative care, satisfaction

## Abstract

**Introduction:**

Telemedicine reduces in-person appointments and extends healthcare services to rural areas. Despite its extended use after the COVID-19 pandemic, further analysis of educational applications and strategies is needed to better prepare parents and caregivers for postoperative pediatric care beyond routine clinical follow-up. Therefore, this review systematically evaluates the effectiveness of telemedicine interventions in educating parents or caregivers after pediatric surgery, with respect to caregiver knowledge and self-efficacy in postoperative care, caregiver satisfaction, and postoperative clinical outcomes.

**Methods:**

Following the PRISMA guidelines, we searched three databases, PubMed, Scopus, and LILACS, for articles published between 2013 and 2023 that involved patients aged 0–18 years who underwent surgery and caregivers who received some form of education through telemedicine. We evaluated the effectiveness of telemedicine for educational purposes by assessing caregiver knowledge, satisfaction, and patient morbidities. Bias was analyzed using the RoB2 and ROBINS-I tools. The certainty of the presented evidence was assessed using the GRADE guidelines. The SWiM guideline was employed to report a structured narrative synthesis from the combined results. The protocol was registered in the International Prospective Register of Systematic Reviews (CRD42024545858).

**Results:**

Four studies were included from 2,163 records initially registered: two randomized controlled trials (RCTs) and two uncontrolled before–after (UCBAs) studies. In the RCTs, caregiver knowledge was significantly higher in the telemedicine intervention group (*p* < 0.05); in one UCBA, caregiver knowledge increased over time. All studies reported high satisfaction with telemedicine, with the RCTs showing significantly higher satisfaction levels than control groups (*p* < 0.05). One UCBA also reported a significant improvement in patient continence. Bias was assessed as moderate in the RCTs and high in the UCBAs. The GRADE criteria indicate a certainty of evidence moderate for satisfaction and caregiver knowledge, and very low for morbidity and rate of complications or adverse events.

**Discussion:**

Telemedicine-based educational tools show promise as a strategy for healthcare systems, achieving high acceptance levels. However, further research is required to refine the methodological approaches for implementing telemedicine in caregiver education within the postoperative setting.

**Systematic review registration:**

https://www.crd.york.ac.uk/PROSPERO/view/CRD42024545858, identifier [CRD42024545858].

## 1 Introduction

The implementation of several technological tools and resources has contributed to strengthening a wide range of aspects associated with health services, from remote attention for routine checks to caregiver-focused education to improve the rehabilitation process (1). Under this approach, the terms telemedicine and telehealth include using medical information delivered via electronic communications to improve the clinical status of the patients (2). Specifically, the remote health approach for rehabilitation is based on providing services for prevention, monitoring, intervention, supervision, and education to overcome geographic, temporal, social, and financial barriers (3). Although the use of basic devices such as telephones for monitoring patients at distance was already implemented around 1960 and medical telehealth as a specific term was established more than 30 years ago (4, 5), the recent COVID-19 pandemic context has significantly piqued the interest in improving and extending the application of these resources. Telemedicine was implemented to avoid exposure to potentially infectious environments and provide continuous care for long-term conditions without in-person encounters (6, 7).

The restrictions associated with the non-pharmaceutical measures taken globally against the SARS-CoV-2 affected the health services offered to vulnerable populations, including pediatric patients. These effects were observed at several levels, such as a decrease in general child life services (8), reduced routine vaccination coverage (9) and postponed non-emergency surgeries (10), together with the classical in-person follow-up appointments (11). One of the critical areas where monitoring and control policies have changed is in the rehabilitation and post-operative care for pediatric patients (12). The high demand for pediatric surgical interventions highlights the need to implement telemedicine for this population. In the USA, approximately 3.9 million pediatric surgeries were performed annually from 2016 to 2019, with 4.7% of children undergoing at least one surgery (13). In lower-middle-income countries, nearly 50% of the population is under 20 years old, indicating a significant need for pediatric surgical services (14). Surgical interventions are crucial for achieving universal health coverage (SDG 3). Insufficient access to safe surgery maintains poverty and hampers economic development, linking to SDGs 1 and 8 (15, 16).

The management related to rehabilitation for these patients includes aspects of patient education, which comprises all materials and strategies for improving literacy and betterment of patient care (17), and high-quality communication focused on Family-Centered Rounds to support decision-making, which increases the involvement of the family during the processes and plan the discharge of the patient for subsequent follow-up and recovery from home (18). The following process of postoperative management at home plays a key role in achieving an adequate recovery and monitoring of multiple aspects associated with surgical trauma, from the commonly expected side effects of postoperative pain (19) to the specific care depending on the surgical intervention. From this point of view, telemedicine has allowed the implementation of dual applications to both educate parents and caregivers to improve rehabilitation management and for a flexible follow-up, which is ideal for rural contexts, to reduce the stress of mobilization to the hospital and, more recently, as valuable tools during the situation of global health emergencies (2, 20, 21).

While various strategies utilizing commercial and customized technologies, as well as applications designed for different devices, have been explored for postoperative follow-up, there is a shortage of studies that consolidate these resources and analyze their use in, specifically, telemedicine focused on education for guiding parents or caregivers during the recovery process of pediatric patients. Based on this, the aim of the present study was to systematically evaluate the effectiveness of telemedicine interventions in educating parents or caregivers after pediatric surgery, with respect to caregiver knowledge and self-efficacy in postoperative care, caregiver satisfaction, and postoperative clinical outcomes.

## 2 Methods

### 2.1 Criteria for study selection

The systematic review was conducted according to the Cochrane Handbook for Systematic Reviews of Interventions (22) and reported following the Preferred Reporting Items for Systematic Reviews and Meta-Analyses (PRISMA) statement (23). The eligibility criteria for study selection were defined *a priori* and applied during the screening process. Studies were included if they were published between 2013 and 2023, involved patients aged 0 to 18 years, and reported surgically treated conditions. The search period was established based on global trends in telemedicine research, highlighting 2013 as an inflection point in the telemedicine literature, with annual mHealth output first exceeding 100 papers and a rapid increase in citations associated with telecare, suggesting a patient-centered telemedicine (24, 25). The primary intervention of interest was the use of telemedicine with an educational focus, delivered through various communication platforms to perform remote interaction. The review included only educational strategies delivered exclusively using telemedicine, with no hybrid components involving in-person clinical care. Eligible interventions had to educate parents or caregivers to support postoperative recovery in pediatric patients. We excluded studies that did not focus on a pediatric postoperative population or that combined telemedicine education with additional clinical interventions. The following study designs were eligible for inclusion: randomized controlled trials (RCTs) and non-randomized controlled trials (NRCTs) where the comparator was a standard not telemedicine-related intervention, uncontrolled before–after (UCBA) studies and interrupted time-series (ITS) with 3 or more data points before and after the intervention. Eligible studies reported at least one of the following outcomes in a quantitative manner: parental or caregiver knowledge, satisfaction with the telemedicine intervention, or reduction in post-surgical morbidity in patients.

Articles were excluded if they focused solely on telemedicine for routine follow-ups or appointments or used a hybrid model combining education and clinical teleconsultation. Additionally, letters to the editor, single case reports, narrative reviews, systematic reviews, meta-analyses, and studies involving patients older than 18 years or with psychiatric, cognitive, or neurological disorders were excluded. Only studies published in English or Spanish were considered. The compliance of all eligibility items was independently checked by three reviewers (G.V.V.C., J.R.R.I., and A.M.P.), followed by revising the full text for the selected articles. Following recommendations of the Cochrane handbook and to ensure transparency in this review, a removal of duplicates was performed after the database search, using the Zotero software. Differences in judgment between reviewers were resolved by consensus through case-by-case discussions among the three authors.

### 2.2 Search strategy and study selection

A systematic literature search was conducted, using PubMed,^1^ renowned for its extensive coverage of health, medicine, and related disciplines, including telemedicine (26); SCOPUS,^2^ which offers broad interdisciplinary coverage (27); and Literatura Latinoamericana y del Caribe en Ciencias de la Salud (LILACS),^3^ which encompasses regional and grey literature to enhance geographical diversity (26). The electronic search strategy was structured in three blocks: population, intervention, and outcomes. The PubMed search string was developed first and then translated to Scopus and LILACS, according to the syntax rules and search manuals for each database. For the case of LILACS, the search was conducted using Spanish terms. The complete search strategies for all three databases are provided in Supplementary Table 1. The studies were retrieved based on selection criteria, checking the title and abstract, and a subsequent evaluation of the full text. The search was concluded on 28/05/2025.

### 2.3 Data management and quality assessment

Data was extracted from the original articles into Excel sheets (Microsoft Windows) and the Cochrane RevMan 7.2.0 version. The generally considered variables were study design, geographical location, application or platform used for interaction with the caregivers, type of surgery, time of measure, questionnaires used, and level of satisfaction with the implemented strategy. The methodological quality of the randomized trials and non-randomized studies was assessed by the Risk of Bias 2 (RoB2) and Risk of Bias in Non-randomised Studies of Interventions (ROBINS-I) tools of Cochrane, respectively (28, 29). All considered results and variables were compiled into a summary table of findings. The methodological approach for explaining and carrying out the telemedicine intervention was also considered. Similarly, the certainty of presented evidence derived from this systematic review was performed by implementing a Grading of Recommendations Assessment, Development and Evaluation (GRADE) approach, based on the Cochrane handbook for grading the certainty of the evidence (30). General data extraction and the implementation of tools for article analysis were carried out by G.V.V.C. and J.R.R.I. Similar to the screening processes, disagreements between the two authors regarding data and bias analysis were resolved through discussion among the three authors.

The implemented protocol for this systematic review was registered and can be accessed on the international database of prospectively registered systematic reviews in health and social care (PROSPERO) under the registration number CRD42024545858.

### 2.4 Synthesis of results

Data synthesis was conducted following the Cochrane Handbook (22) and the SWiM guideline (Synthesis Without Meta-analysis) (31). Studies were grouped *a priori* by design and, within each design, by outcome: caregiver knowledge, satisfaction, and postoperative complications and benefits in the quality of life of patients. Because methodological heterogeneity ruled out a meta-analysis, a structured narrative synthesis with vote counting based on the direction of the point estimate was applied. An upward arrow (↑) was assigned when the point estimate favored tele-medicine, a downward arrow (↓) when it favored the comparator, and a horizontal arrow (↔) when the direction was unclear. Effect estimates are presented in their original units. *p*-values are reported for context only.

Heterogeneity was explored qualitatively by comparing population characteristics, intervention components and risk-of-bias judgments across studies. Certainty of the evidence was assessed with the GRADE framework and summarised. No study or outcome was prioritised. All results received equal interpretative weight because each addressed the evaluated outcomes. The results were presented in a summary table describing the characteristics of the included studies, as well as in an additional table showing the certainty of the evidence for each outcome analyzed. Limitations of this synthesis approach are discussed in the Discussion section.

### 2.5 Appraisal of included studies

Ethics committee approval was not required as this manuscript is a systematic review article.

## 3 Results

**Figure 1:**
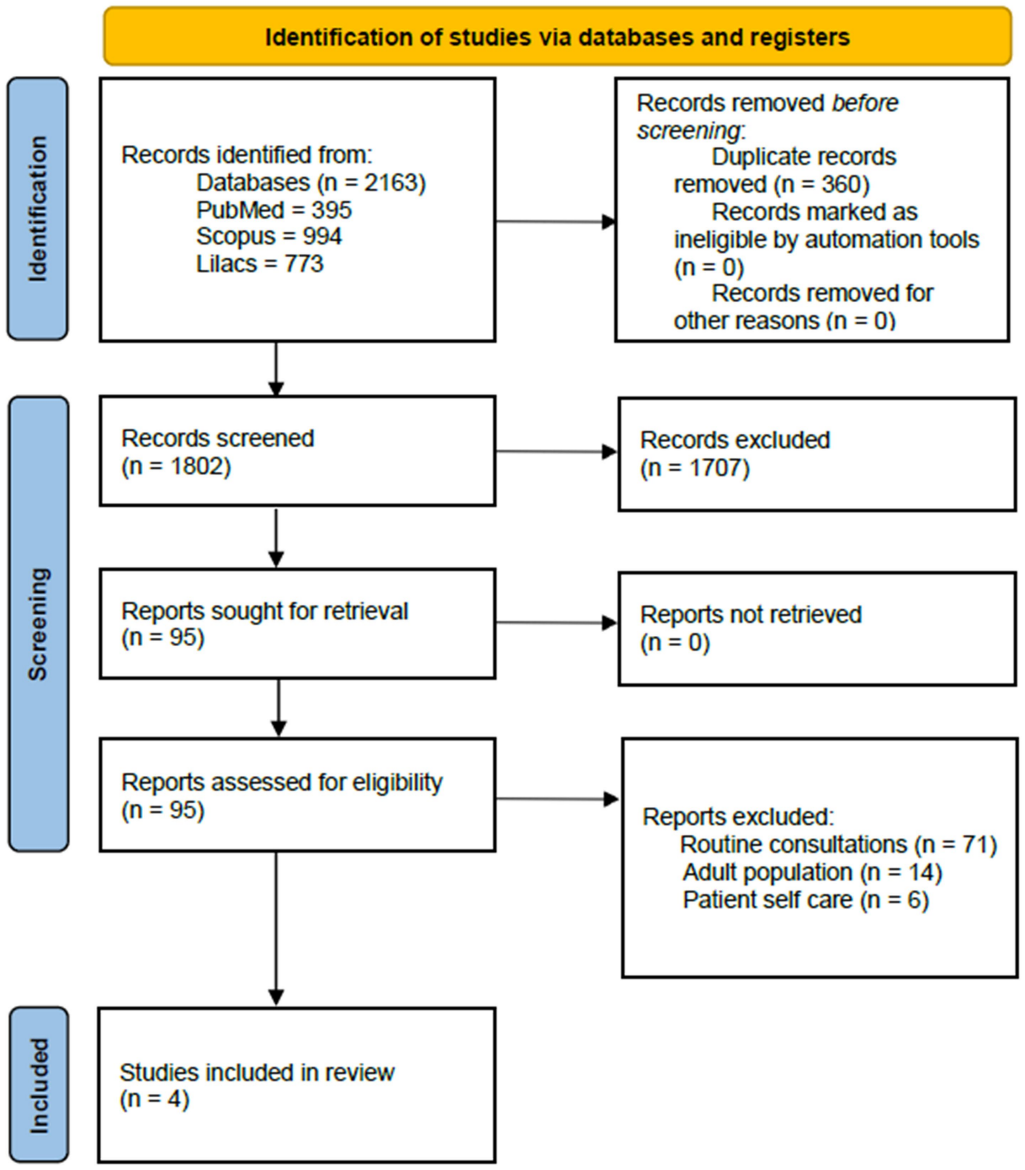
PRISMA flow diagram.

The initial searches yielded 994 studies in Scopus, 395 in PUBMED, and 773 in LILACS, for 2,163 records registered at the identification stage. After excluding the duplicates and a subsequent title and abstract screening, 95 papers remained for full-text review. An additional 91 studies were excluded during the full-text review, considering the inclusion criteria and the specificity of the educational purpose, and no hybrid interventions (telemedicine and in-person), or interventions with purposes other than educational, such as those focused on providing patient care. Therefore, most of the articles excluded at this stage were studies on telemedicine for routine consultations. A smaller proportion involved adult populations or interventions aimed at patient self-care. The final review comprised 4 articles, consisting of 2 UCBA (32, 33) and 2 RCTs studies (34, 35), published between 2020 and 2022 (Figure 1).

### 3.1 General characteristics of included studies

The population assessed in the included studies varied from 2 to 74 participants, with follow-up durations ranging from 1 to 4 months and documenting four types of surgical conditions. Among the platforms employed for remote health aimed at education, the WeChat app was used in the 2 RCTs, and only one study indicated the use of mixed tools, including video and phone calls. The comparator (control groups for RCT) utilized information provided through manuals and leaflets. The resources for reporting the main outcomes were also diverse, with several surveys used to estimate caregiver knowledge. Two studies employed the Patient Satisfaction Questionnaire Short Form 18 (PSQ-18) for satisfaction quantification. Additionally, from the four included studies, only Yu et al. offered a more detailed explanation of how the telemedicine intervention was implemented (34). This was based on the WeChat platform and consisted of an attending physician and two nursing supervisors to distribute information on breastfeeding, including its importance, operational skills, and hygiene practices. A summary of the main criteria and extracted data from the included works is provided in Table 1.

**Table 1:** Characteristics of the studies included in the revision (*n* = 4).

Study	Type of study	Participants and time of the study	Type of surgical intervention	Remote health tools used and comparators	Type of outcomes	Outcomes and tools of evaluation	Main findings	Risk of bias
Knaus et al. (32)	Uncontrolled before-after studies.	*n* = 67. Average age of 8.6 years. Follow-up: 3 months.	Bowel management associated with anorectal malformations, Hirschsprung’s disease, myelomeningoceles and spinal injury.	Video and phone calls.	Complications, quality of life and caregiver and satisfaction.	PedsQL, BCS, VSS and parent/patient satisfaction survey.	No significant difference (*p* = 0.70) was detected in PedsQL scores between discharge and three months, with ↓80.9 vs. 82.4 values. Both the BCS and the VSS improved markedly after the telemedicine intervention: BCS fell from 28.2 at baseline to ↑17.2 at one month and ↑12.7 at three months, and VSS declined from 14.2 to ↑9.3 and ↑8.7 over the same intervals (*p* < 0.01 for both baseline comparisons). Sixty-two percent of parents completed the satisfaction survey, with a median score of ↑5 (very satisfied) for all questions. Over ↑75% of parents said they would prefer a telemedicine program over an in-person program. No declaration was found about the missing data on excluded participants.	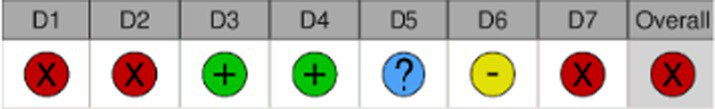
Moreno and Peck (33)	Uncontrolled before-after studies.	*n* = 2, classified as pediatric. Follow-up: 4 weeks.	Tracheostomy.	Telehealth.	Knowledge of caregiver and satisfaction.	CKC, CTMS and TSS.	Knowledge increased each time the checklist was given (2 times), achieving the intended score goals of ↑ > 8 for CKC and ↑ > 25 for CTMS. Caregiver satisfaction with the telehealth experience exceeded the minimum score of ↑14.	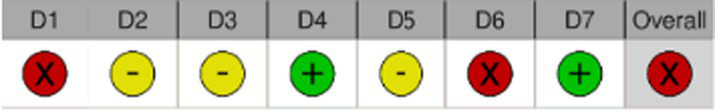
Yu et al. (34)	Randomized control trial.	*n* = 30. Average of 2.1 months. Follow-up: 3 months.	Cardiac surgery.	WeChat and phone calls. Comparator: manual.	Knowledge of caregiver, satisfaction and complications	BSES and the PSQ-18. Adverse events (feeding intolerance, abdominal distension, dyspeptic diarrhea, weight gain, recurrent vomiting).	BSES scores in the telemedicine group increased from 86.4 ± 13.5 at discharge to ↑139.4 ± 15.8 at three months and were significantly higher than those in the control group (*p* = 0.000). The telemedicine group achieved a higher rate of exclusive breastfeeding at three months (↑73.3% vs. 46.7%, *p* = 0.035). BSES scores within the control group did not change over time (*p* = 0.756). PSQ-18 satisfaction score was greater in the telemedicine group than the control group (↑3.92 vs. 3.72, *p* < 0.001). Adverse events: No statistical differences between groups. No statistical differences between groups (*p* values from 0.150 to 0.585).	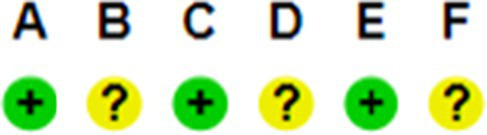
Zhang et al. (35)	Randomized control trial.	*n* = 74. Average of 2.4 years. Follow-up: 1 month.	Ventricular septal defects.	WeChat. Comparator: leaflet.	Knowledge of caregiver, satisfaction and complications.	CKQ and the PSQ-18. Postoperative complications (pulmonary infections, arrhythmias, poor incision healing, occluder detachment).	The WeChat group showed a significantly higher CKQ score than the leaflet group (↑68.8 ± 8.9 vs. 46.4 ± 10.5, *p* = 0.010). In the PSQ-18, the WeChat group scored higher in general satisfaction ↑4.7 ± 0.8 vs. 3.2 ± 1.2 (*p* = 0.041), interpersonal manner ↑4.6 ± 0.5 vs. 2.8 ± 0.9 (*p* = 0.033), communication ↑4.8 ± 0.9 vs. 2.9 ± 1.0 (*p* = 0.027), time spent with the physician ↑4.9 ± 0.6 vs. 2.8 ± 1.3 (*p* = 0.013), and accessibility/convenience ↑4.9 ± 0.8 vs. 3.0 ± 1.1 (*p* = 0.020). No differences were observed for technical quality ↑4.3 ± 0.9 vs. 4.2 ± 0.7 (*p* = 0.902) or financial aspects ↓4.1 ± 0.7 vs. 4.2 ± 1.1 (*p* = 0.879). Postoperative complications did not differ between groups ↔2 vs. 2 (*p* = 0.447).	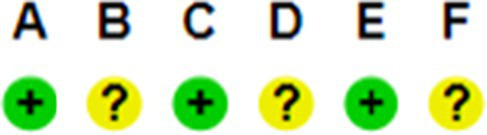

*Domains of bias in ROBIS-I.* D1: Bias due to confounding. D2: Bias in the selection of participants in the study. D3: Bias in the classification of interventions. D4: Bias due to deviations from intended interventions. D5: Bias due to missing data. D6: Bias in measurement of the outcome. D7: Bias in the selection of the reported result. Judgment of risk: Serious (red). Moderate (yellow). Low (green). No information (blue).

*Domains of bias in RoB2*. A: Bias arising from the randomization process. B: bias due to deviations from intended interventions. C: bias due to missing outcome data. D: bias in measurement of the outcome. E: bias in the selection of the reported result. F: overall bias. Judgment of risk: Some concerns (yellow). Low (green).

Vote counting based on the direction of the point estimate: ↑ Favored tele-medicine. ↓ Favored the comparator. ↔ Unclear direction of the effect.

CKC, Caregiver Knowledge Checklist; CTMS, Care Transitions Measure Survey; BSES, Breastfeeding Self-Efficacy Scale; CKQ, Caretaker Knowledge Questionnaire; TSS, Telehealth Satisfaction Survey; PSQ-18, Patient Satisfaction Questionnaire Short-Form; PedsQL, Pediatric Quality of Life Inventory; BCS, Baylor Continence Scale; CCCS, Cleveland Clinic Constipation Score; VSS, Vancouver Symptom Score for Dysfunctional Elimination Syndrome.

### 3.2 Risk of bias assessment

Among the analyzed randomized trial studies, there were some concerns about the design of the studies, as overall judgment. Specifically, Yu et al. (34) and Zhang et al. (35) raised concerns about the risk of bias due to the potential participant awareness of their assignment during the trial, impacting two domains of bias. In the case of non-randomized studies, UCBAs studies, the general risk of bias was serious. Knaus et al. (32) and Moreno and Peck (33) demonstrated a serious risk due to a lack of adjustments for confounding factors related to patient or caregiver baseline characteristics. Additionally, other classifications of serious risks or biases included participant awareness of the intervention received (33) and the lack of equivalence in the analysis applied to intervention groups (32). One study was classified as “No Information” in one of the domains due to a need for more information about missing data on excluded participants (Table 1).

### 3.3 Certainty of evidence

Due to the heterogeneity of the studies, not all retrieved articles were included to evaluate the certainty of each outcome. The level of satisfaction with the intervention was assessed in all four included studies. Knowledge level was evaluated in three of them (33–35), while complications and benefits in quality of life were assessed in three studies as well (32, 34, 35). Table 2 shows the results of applying the GRADE tool for judgment of the certainty of evidence. Morbidity is downgraded by the inconsistency, indirectness, and imprecision criteria. This is due to mixed results, where Knaus et al. report favorable outcomes, while the other studies do not report significant results with the telemedicine intervention. Additionally, different clinical outcomes are reported between the four studies, and imprecision is due to the inconsistent reporting of confidence intervals in the two RCTs. Moreover, imprecision impacts the satisfaction and knowledge outcomes because a small “n” of participants reported in one study. The risk of bias also affects all outcomes. After applying the GRADE criteria, the certainty of evidence is moderate for satisfaction and knowledge, and very low for morbidity.

**Table 2:** Evaluation of the certainty of evidence using the GRADE tool.

Outcome	Evaluated effect	Participants in each study	Certainty judgement	Reasons for downgrading
Parent and/or caregiver satisfaction.	Telemedicine probably improves satisfaction. All included studies report higher levels of satisfaction with the intervention.	173 (4 studies).	Moderate.	RoB and imprecision.
Caregiver knowledge.	Telemedicine probably increases caregiver knowledge scores.	106 (3 studies).	Moderate.	RoB and imprecision.
Complication rate and patient morbidities.	Evidence is uncertain about the effect on clinical complications. Reports of mixed results, inconsistency of CIs described and different clinical outcomes measured.	171 (3 studies).	Very low.	RoB, inconsistency, indirectness, and imprecision.

CI, confidence intervals; RoB, Risk of bias.

### 3.4 Main outcomes analyzed

For the analysis of the various elements evaluated in this systematic review, the authors of the included studies used a series of standardized questionnaires. These included: The Caregiver Knowledge Checklist (CKC), used to assess caregivers’ knowledge. The Care Transitions Measure Survey (CTMS) evaluates caregiver preparedness during the care transition process. The Breastfeeding Self-Efficacy Scale (BSES), designed to measure the confidence of the mother in her ability to breastfeed. The Caretaker Knowledge Questionnaire (CKQ) indicates the level of knowledge of the caregiver. The Telehealth Satisfaction Survey (TSS), used to assess satisfaction or dissatisfaction with telehealth interventions. The Patient Satisfaction Questionnaire Short-Form (PSQ-18) measures multiple dimensions of patient satisfaction, including general satisfaction, technical quality, interpersonal manner, communication, financial aspects, time spent with the physician, accessibility, and convenience. The Pediatric Quality of Life Inventory (PedsQL), a tool designed to measure health-related quality of life in children and adolescents. The Baylor Continence Scale (BCS) assesses the impact of fecal incontinence on quality of life in pediatric populations. The Cleveland Clinic Constipation Score (CCCS), also known as the Wexner Constipation Score, quantifies the severity of chronic constipation in both adults and children. The Vancouver Symptom Score for Dysfunctional Elimination Syndrome (VSS), used to identify symptoms of dysfunctional elimination syndrome in children, including both urinary and defecatory problems.

#### 3.4.1 Parent and caregiver knowledge

Moreno and Peck used the CKC, which includes items for evaluating tracheostomy management skills (33). A CTMS was also used in this study to evaluate the preparedness of the caregiver. These surveys were applied during the 2-week telehealth visit and in a face-to-face visit 4 weeks later. The results indicated that knowledge increased each time the checklist was given, achieving the intended score goals of >8 for CKC and >25 for CTMS. The other UCBA study, Knaus et al. did not assess the level of prior knowledge of the subject (32).

Yu et al. applied the BSES, which contained 33 items in total, with a 5-point Likert scale, associated with skill and psychological dimensions, for measuring the breastfeeding self-efficacy, after congenital cardiac surgery (34). The knowledge measured by this tool was similar among the evaluated groups at discharge. However, during the 3-month follow-up, the BSES in the telemedicine group increased markedly, from 86.4 ± 13.5 at discharge to 139.4 ± 15.8 points, whereas the control group showed no significant change, from 85.5 ± 9.7 to 92.3 ± 9.4. Consequently, at 3 months postdischarge, the telemedicine group demonstrated significantly (*p* = 0.00) higher breastfeeding self-efficacy and greater confidence in caring for their infants than the standard care group.

In the case of the second RCT study conducted by Zhang et al., the CKQ is a survey comprising 18 items, with possible scores ranging from 0 to 4 points (35). Different score ranges indicate the level of knowledge: a score < 44 is classified as a low knowledge level, 44 to 58 is classified as an intermediate knowledge level, and > 58 is classified as a high knowledge level. The reported CKQ scores in the WeChat group (68.8 ± 8.9) were significantly higher than those in the leaflet group (46.4 ± 10.5) during the perioperative period.

#### 3.4.2 Patient satisfaction

The satisfaction survey used by Knaus et al. consisted of 10 questions evaluated using a Likert scale ranging from 1 (very dissatisfied) to 5 (very satisfied) (34). Additionally, the survey included multiple-choice queries and a section for free-text comments. The results describe 91% (39/43) of parents indicating that the telemedicine appointments were less stressful for their child compared to in-person visits, and over 75% (33/43) of parents said they would choose a telemedicine program over an in-person program. Additionally, over 75% of parents said they would choose a telemedicine program over an in-person program. In Moreno and Peck study, the TSS consisted of 10 questions with dichotomous responses of satisfied or unsatisfied and a result of >14 as an intended score goal (33). At each patient encounter, caregivers expressed gratitude for ongoing communication and continuity of quality care.

The PSQ-18, employed in the two analyzed RCTs, is a validated form comprising 18 questions. It utilizes a five-point Likert scale (1–5) to measure various dimensions, including general satisfaction, technical quality, interpersonal manner, communication, financial aspects, time spent with the physician, accessibility, and convenience. Yu et al. reported significantly higher satisfaction in the study group (*p* < 0.001), compared to the control group (34). In this study, all dimensions analyzed in the survey were significantly higher in the remote health intervention group. Similarly, Zhang et al. reported higher general satisfaction in the WeChat group than in the leaflet group and described better satisfaction in five out of seven dimensions using the app (35).

#### 3.4.3 Complication rate

Knaus et al. applied the PedsQL, and PROMs were collected for each patient at the first clinic visit before the program (intake), at the 1-month follow-up, and at the 3-month follow-up after the BMP (34). The recorded PROMs included the BCS, CCCS, and the VSS. Before the BMP, stool continence data were available for 42 patients aged 4 or older. Of these patients, 28.6% (12/42) were continent of stool at intake. At the 1-month follow-up, 45.1% (23/51) were continent of stool, and at the 3-month follow-up, 68.9% (31/45) were continent of stool (*p* = 0.1 and *p* < 0.01, respectively). Before the BMP, urine continence data were available for 47 patients, and 55.3% (26/47) were continent of urine at intake. There was a significant improvement in the BCS and VSS scores after undergoing the telemedicine BMP (*p* < 0.01 for 1-month and 3-month follow-up compared to baseline). Urinary continence improved to 63.6% (28/44) at the 1-month follow-up, with further improvement to 73.7% (28/38) at the 3-month follow-up, although these differences were not statistically significant (*p* = 0.42 and *p* = 0.09, respectively).

Zhang et al. mention no significant difference between the two groups in postoperative complications (such as pulmonary infections, arrhythmias, poor incision healing, residual shunts, and occlude detachment) (35). Yu et al. describe that there was no significant difference in the incidence of feeding intolerance, abdominal distension, dyspeptic diarrhea, weight gain, or recurrent vomiting between the two groups during the 3-month follow-up period (*p* > 0.05) (34). The prevalence of these adverse events was smaller in the study group. Moreno and Peck did not evaluate morbidity or complications.

## 4 Discussion

Communication technologies have advanced for decades, presenting an unprecedented opportunity to overcome the limitations imposed by traditional medical services, where both the provider and the recipient must be physically present in the same location simultaneously. Information and communication technologies, including telemedicine, are pivotal for improving healthcare services (36).

Interestingly, most of the published articles on telemedicine for pediatric patients are focused on the postoperative classical follow-up rather than specifically on education for caregivers. In addition to the PRISMA screening applied in our research to exclude studies involving the typical use of telemedicine for routine checks, this aspect can also be noted in a recent systematic review of pediatric orthopedic trauma patients. This review assessed the satisfaction of both patients and surgeons with telemedicine remote care during the COVID-19 pandemic, as reported in the included articles (37). On the other hand, our systematic review found a few studies that specifically analyze telemedicine focused on the education of caregivers about postoperative care. Overall, the data we have reviewed suggests that implementing a remote health approach can improve the knowledge of caregivers to attend to the patients at home, with increased satisfaction compared to the use of manuals and documents for education, and also decreasing the complication rates.

However, when analyzing the mechanism for carrying out parental and caregiver education in the postoperative environment, there is a lack of detailed information for explaining the designed and implemented methodological approach, such as the use of videos, apps, the employment of management guides, and simulation strategies, among others. This hinders the efficient replication of this type of intervention by medical groups interested in applying this method. It also does not allow for assessing the potential and effectiveness of these strategies in achieving caregiver education. One of the studies in our review describes the use of dedicated personnel and the WeChat app to educate parents and share experiences in the postoperative context (34). Beyond the articles reviewed here, other studies have suggested developing specific instructional programs. These programs, supported by the WeChat app, would provide content on basic and advanced knowledge regarding patient conditions and how to conduct appropriate care procedures to prevent infections, using clear and understandable language (38). Furthermore, this social media tool has been described in China for general patient care education in several areas related to stroke patients (39) and coronary artery disease (40).

Regarding the bias analysis conducted in this systematic review, there is potential for improvement in both RCT and UCBA studies, particularly in clearly defining the groups receiving the telemedicine intervention and those that do not, as well as establishing the patient baseline information, which is primarily lacking in non-randomized studies. Other serious risks or biases classifications included the participant awareness of the intervention received and the lack of equivalence in the analysis applied to the intervention group. Including information about the studied population, such as age group, the level of education and knowledge of the caregiver, and easy access to technological tools, may enable the creation of more homogeneous groups for comparison or enhance the before-and-after study approach. Regarding the certainty of the presented evidence analysis, moderate certainty evidence suggests that the telemedicine interventions improve the knowledge of the caregivers about postoperative care in the home setting and increase their overall satisfaction with the intervention. These findings indicate that telemedicine can provide parents with additional resources to manage the postsurgical needs of patients and may promote a wider adoption of such programs. However, the evidence for clinical morbidity and complications is of very low certainty. Therefore, we cannot determine whether telemedicine reduces postsurgical complications or adverse postoperative events that require a classical assessment. Further well designed trials with uniform outcome measures are needed before firm conclusions can be drawn about safety and clinical effectiveness.

To assess caregivers satisfaction with telemedicine programs, the validated PSQ-18 survey was employed, which also allowed for determining the general satisfaction, technical quality, interpersonal manner, communication, financial aspects, time spent with the physician, accessibility, and convenience, providing more comprehensive information about the various elements involved in the satisfaction of a program, not just a single value, but enabling the determination of areas for improvement in future research. Patient satisfaction is becoming increasingly important in healthcare, encompassing numerous aspects that warrant evaluation. Valdes et al. conducted a study to determine if the PSQ-18 was an appropriate outcome measure for assessing patient satisfaction among individuals undergoing hand therapy (41). The findings suggest that the PSQ-18, when adapted for hand therapy, is a suitable outcome measure for assessing patient satisfaction in this context, as it evaluates multiple subscales of the phenomenon. In the case of UCBA studies, they focused exclusively on providing numerical information regarding the overall satisfaction of the caregiver. They did not focus on the social component linked to patient care, represented by economic, communicative, and accessibility aspects. The aspects assessed in these scales include important variables that must be considered when selecting a telemedicine or in-person program.

Regarding postoperative complications and morbidity, despite not obtaining statistically significant results in 2 RCTs studies where this variable was evaluated, it can be highlighted that with the support of the intestinal management program, the percentage of digestive incontinence decreased progressively during follow-up, which was statistically significant for Knaus et al. (32).

It is important to highlight that telemedicine is subdivided into different areas, with teleassistance being the most widely developed in the scientific literature. Its benefits regarding the reduction of space–time variables are clear. However, the area of telemedicine aimed at education for caregivers has not been as extensively developed. The literature reflects its utility more for educational purposes within the medical community, rather than directed towards mothers, fathers, and caregivers, which still needs to be explored. The applicability of telemedicine includes an important social component, which can be evidenced in this systematic review. For example, despite the lack of statistically significant differences in complications or morbidity between groups in some of the studies, treated in person and those managed via telemedicine, there is evident satisfaction among caregivers regarding telemedicine programs for educational purposes.

Factors such as the availability of staff, educational materials, and savings in terms of travel or mobility potentially contribute to user satisfaction. As healthcare professionals, we understand how overwhelming a hospital admission can be, and doubts about managing a particular condition often arise at home when we do not have the presence of healthcare personnel. Having the educational support of healthcare professionals, or their availability to address any doubts that may arise at home, is a subjective but highly relevant variable provided by remote practices. An example of this is the statistically significant improvement in continence observed in patients enrolled in intestinal management programs. Yang et al. conducted a systematic review to assess the effects of telemedicine on the burden, anxiety, depression, and quality of life of informal caregivers of patients receiving palliative care (42). They included 9 randomized controlled trials and concluded that telemedicine statistically significantly reduced the caregiving burden and anxiety of informal caregivers. However, it had no significant effect on depression or quality of life.

Beyond clinical outcomes, the implementation of telemedicine related to pediatric postsurgical care also carries implications for optimizing sustainability within healthcare structures. Maia et al. (43) described a Pediatric Telecardiology Service in Portugal, whose implementation resulted in cost savings of approximately 1.1 million euros and 419 euros per patient for the healthcare system. Other studies emphasize three fundamental pillars of telemedicine sustainability: cost reduction, improved service utilization, and enhanced quality of care. These aspects align directly with Sustainable Development Goals (SDGs) 3 and 10, promoting well-being and reducing inequalities (44). Achieving SDGs related to telemedicine also involves overcoming challenges to facilitate broader adoption. Critical factors include robust digital infrastructure, sustainable financing models supported by public-private partnerships, and continuous professional training (45).

The main limitation of this review was the inability to conduct a quantitative synthesis or meta analysis. Despite the creation of a summary of findings table and assessing certainty with GRADE, substantial methodological heterogeneity, including differences in study design, outcome instruments, and the outcomes themselves, prevented statistical pooling and reduced the overall certainty of the evidence. Another potential limitation was the heterogeneity of outcome metrics across studies. To address this, we report the exact *p*-values and present each result in the original units of its measurement tool, therefore strengthening the narrative synthesis, supported by the vote counting method. Nonetheless, the findings of this study suggest the advantages and satisfaction of telemedicine for the education of caregivers, considering the context in which it is applied and the general necessity for well-described methodologies for training parents and caregivers. Similarly, the implementation of the remote health education process in postoperative care is highly dependent on the context, given potential limitations related to access to technology. This necessitates the use of platforms and resources based on the availability of devices. Consequently, changes need to be instituted through regulations and policies that incorporate this new service modality centered on education, ensuring its authorization by insurers (46).

Although there are few publications about the use of telemedicine for educational purposes in the field of pediatrics, specifically in pediatric surgery, this approach is increasingly common in adult care, due to the public health benefits these interventions can offer. A cluster-randomized controlled trial conducted in Guangzhou, China, showed that a six-month self-management intervention, including health education, health promotion, group chat, and blood pressure monitoring, and delivered via WeChat, could feasibly and effectively help patients reduce blood pressure and improve self-management effectiveness (47). Digital health has shown its reach not only in the context of chronic diseases and surgical conditions, but also in the development of strategies such as reducing tobacco consumption. For example, the study published by Cupertino et al. (48) *Vive Sin Tabaco. ¡Decídete!,* a web-based (e-health) decision aid designed to help Mexican smokers create a quit plan and access cessation resources. The intervention included 164 smokers from two primary care clinics who presented with both physical and psychological dependence on nicotine. Two out of ten smokers who used the tool successfully quit smoking, with a 3-month follow-up rate of 81.5% and a 7-day point prevalence abstinence rate of 19.1%.

Lastly, from a prospective point of view, this review was used as a starting point for developing a telemedicine program aimed at mothers, fathers, and caregivers of pediatric post-surgical patients with gastrostomies, ileostomies, and colostomies in the provinces of Pichincha, Guayas, and Manabí in the South American country of Ecuador. The goal is to provide tools to caregivers for vulnerable populations who may benefit from telemedicine follow-up for educational purposes due to socioeconomic, geographical, and even healthcare system saturation issues. This program is intended to be delivered by healthcare professionals, and it aims to reduce patient morbidity, seeking to contribute to the scientific literature in an area that is currently under-researched.

## 5 Conclusion

The results showing increased caregiver knowledge and higher satisfaction with the remote approach indicate that telemedicine tools represent a promising strategy for patients and healthcare systems in the education and care of postoperative pediatric patients. Despite the intrinsic limitations, it is a valid strategy that adapts to the day-to-day lives of patients and their caregivers. However, more studies with detailed explanations of the methodological forms are needed to adjust the telemedicine strategy, reduce biases and reinforce the attention of government policies to facilitate its implementation in society beyond remote care, favoring the utilization of the tool for educational purposes.

## Data Availability

The datasets presented in this study can be found in online repositories. The names of the repository/repositories and accession number(s) can be found in the article/Supplementary material.

## Footnotes

^1^ https://pubmed.ncbi.nlm.nih.gov/

^2^ https://www.scopus.com/home.uri

^3^ https://lilacs.bvsalud.org/es/
