# Supplementary material for: Telemedicine for educating parents or caregivers for postoperative care of pediatric patients: a systematic review
Source: Front Public Health. 2025 Jul 30;13:1606211. doi: 10.3389/fpubh.2025.1606211 (PMC12343499; doi:10.3389/fpubh.2025.1606211)
Supplement: Supplementary file 1 [file Table_1.docx]

***Supplementary Material***

# Supplementary Table 1

| **Database** | **Search lines** |
| --- | --- |
| PubMed | (pediatric*[tiab] OR child*[tiab] OR adolescen*[tiab] OR infant*[tiab])  AND (Telemedicine[Mesh] OR "Remote Consultation"[Mesh] OR telemedicin*[tiab] OR telehealth*[tiab] OR "tele-health"[tiab] OR ehealth[tiab] OR mhealth[tiab] OR "m-health"[tiab] OR telecare[tiab] OR "mobile app*"[tiab] OR smartphone*[tiab] OR wechat*[tiab] OR "we chat"[tiab])  AND ("follow-up"[tiab] OR followup[tiab] OR postoperative*[tiab] OR "post-operative"[tiab] OR perioperat*[tiab] OR aftercare[tiab] OR discharge[tiab] OR "post-discharge"[tiab] OR "management program"[tiab]) AND  (satisfaction[tiab] OR "patient satisfaction"[tiab] OR "caregiver satisfaction"[tiab] OR "family satisfaction"[tiab] OR experience[tiab]) AND ("2013/01/01"[PDAT] : "2023/12/31"[PDAT]) |
| SCOPUS | TITLE-ABS-KEY (pediatric* OR child* OR adolescen* OR infant*) AND TITLE-ABS-KEY ("Telemedicine" OR "Remote Consultation" OR telemedicin* OR telehealth* OR "tele-health" OR ehealth OR mhealth OR "m-health" OR telecare OR "mobile app*" OR smartphone* OR wechat* OR "we chat") AND TITLE-ABS-KEY ("follow-up" OR followup OR postoperative* OR "post-operative" OR perioperat* OR aftercare OR discharge OR "post-discharge" OR "management program") AND TITLE-ABS-KEY (satisfaction OR "patient satisfaction" OR "caregiver satisfaction" OR "family satisfaction" OR experience) AND PUBYEAR > 2012 AND PUBYEAR < 2024 |
| LILACS | (pediatr* OR niñ* OR adolescen* OR lactant*)  AND (mh:Telemedicina* OR mh:"Consulta Remota"* OR telemedicin* OR telesalud* OR "tele-salud" OR e-salud OR m-salud OR "m-salud" OR telecuidad* OR "aplicación móvil*" OR "teléfono inteligente*" OR wechat* OR "we chat")  AND ("seguimiento" OR seguimient* OR postoperatori* OR "post-operatori*" OR perioperatori* OR cuidadoposterior* OR alta* OR "post-alta" OR "programa de manejo*" OR "programa de gestión*") AND  (satisfacción OR "satisfacción del paciente" OR "satisfacción del cuidador" OR "satisfacción familiar" OR experiencia)  AND (year_cluster:2013 OR year_cluster:2014 OR year_cluster:2015 OR year_cluster:2016 OR year_cluster:2017 OR year_cluster:2018 OR year_cluster:2019 OR year_cluster:2020 OR year_cluster:2021 OR year_cluster:2022 OR year_cluster:2023) |
